# Genetic association of *TOLLIP* gene polymorphisms and HIV infection: a case-control study

**DOI:** 10.1186/s12879-021-06303-4

**Published:** 2021-06-21

**Authors:** Ming-Gui Wang, Jing Wang, Jian-Qing He

**Affiliations:** 1 Department of Respiratory and Critical Care Medicine, Clinical Research Center for Respiratory Disease, West China Hospital, Sichuan University, No. 37, Guo Xue Alley, Chengdu, 610041 Sichuan Province People’s Republic of China; 2 Department of Infectious Disease, Neijiang Second People’s Hospital, Neijiang, Sichuan Province People’s Republic of China

**Keywords:** Susceptibility, Toll interacting protein, HIV, Single nucleotide polymorphism, Genetic

## Abstract

**Background:**

Previous studies have indicated that host genetic factors play an essential role in immunity to human immunodeficiency virus (HIV) infection. We aimed to investigate the association between the toll-interacting protein (*TOLLIP*) and mannose-binding lectin 2 (*MBL2*) genes and HIV infection susceptibility among Chinese Han patients.

**Methods:**

This is a case-control study. A total of 435 HIV-infected patients and 1013 seronegative healthy individuals were recruited. DNA was extracted from whole blood. Two SNPs in the *MBL2* gene (rs7096206 and rs1800450) and three SNPs in the *TOLLIP* gene (rs5743899, rs3750920, and rs5743867) were selected and genotyped using a SNPscan Kit (Cat#: G0104, Genesky Biotechnologies Inc., Shanghai, China). Odds ratios (ORs) and their 95% confidence intervals (CIs) were calculated using unconditional binary logistic regression.

**Results:**

A significant association between the minor alleles rs5743899 (C allele) and rs5743867 (G allele) in the *TOLLIP* gene and susceptibility to HIV infection was found in this study after adjusting for age and sex (P^a^ = 0.011 and < 0.001, respectively). The rs5743867 in the *TOLLIP* gene was significantly associated with the risk of HIV infection in dominant, recessive, and additive models when adjusted for age and sex (P^a^ < 0.05). No significant association was found between *MBL2* gene polymorphisms and HIV infection.

**Conclusion:**

Our study found a statistically significant association between the two SNPs (rs5743867 and rs5743899) in the *TOLLIP* gene and susceptibility to HIV infection in a Chinese Han population.

**Supplementary Information:**

The online version contains supplementary material available at 10.1186/s12879-021-06303-4.

## Background

Acquired immune deficiency syndrome (AIDS) is a condition caused by human immunodeficiency virus (HIV), a virus that progressively destroys the immune system. This remains a major worldwide public health problem. There were 1.7 million new HIV-infected patients worldwide, and 0.69 million died from AIDS-related illnesses in 2019 [1]. New HIV infections have declined by 23% compared to 2010 [1]. In 2019, 67% of all people living with HIV were receiving antiretroviral therapy. It is well known that the risk of acquiring HIV is higher among certain populations: men who have sex with men, people who inject drugs, sex workers and transgender people [1]. Currently, host genomics has attracted much attention, and considerable evidence has demonstrated that genetic factors play an essential role in HIV infection [2, 3].

Data on host genetics have broadened our understanding of host factors’ importance in susceptibility to HIV infection [2, 3]. Recent studies have indicated that several important host polymorphisms play a vital role in HIV infection and the progression to AIDS [2–5]. Human leukocyte antigen (HLA) type is a widely studied example of host factors in the course of HIV [4, 5]. Another important host factor polymorphism is the CCR5 deletion mutation CCR532, which is the only genotype that has been consistently identified as protective against HIV infection [4, 6]. Researchers have also found that the toll-interacting protein (*TOLLIP*) and mannose-binding lectin 2 (*MBL2*) genes may also play a vital role in HIV infection and the progression to AIDS [7–10].

The *TOLLIP* gene encodes a ubiquitin-binding protein that interacts with several Toll-like receptor (TLR) signaling cascade components. TLR signaling has been widely suggested to inhibit HIV and other retrovirus infections [11–13]. A previous study found that the rs5743867 polymorphism is significantly associated with protection from sepsis [14], and the GG genotype of rs5743867 was associated with increased risks for pulmonary tuberculosis [15]. In a case-population study in Vietnam, researchers found that SNPs rs5743899 and rs3750920 in the *TOLLIP* gene were associated with susceptibility to tuberculosis, which demonstrates that *TOLLIP* deficiency is associated with an increased risk of infectious disease [16]. In addition, rs3750920 was associated with decreased levels of *TOLLIP* mRNA expression, and rs5743899 was associated with increased IL-6 production [16]. The host factor *TOLLIP* gene, which is involved in TLR signaling, may play an important role in HIV infection. Researchers found that the *TOLLIP* gene plays a crucial role in inhibiting HIV infection and regulating the incubation period of the virus [7]. Others found that the *TOLLIP* gene suppressed NF-κB-dependent HIV-1 TLR-driven transcription, which indicates the potential role of the genetic factor *TOLLIP* in maintaining viral persistence [17].

The *MBL2* protein can recognize and bind to mannose and N-acetylglucosamine on many microorganisms, including bacteria, yeast, and viruses such as influenza and HIV virus [18]. Therefore, polymorphisms of the *MBL2* gene may be related to susceptibility to autoimmune and infectious diseases. When compared with healthy controls, a meta-analysis found that *MBL2* exon 1 polymorphisms were associated with host susceptibility to HIV-1 infection in three genetic models (dominant, recessive and allelic model) (*P* < 0.05), and the *MBL2* 0/0 mutant allele has also been previously associated with an increased risk for HIV infection (*p* < 0.00001) [19]. Subgroup analysis by ethnicity showed that significantly elevated risks were found in Caucasians in the recessive model but not in Asians [19]. In addition, variants in the *MBL2* gene have also been shown to be related to disease progression to AIDS and death [20, 21]. When comparing the frequency of *MBL2* promoter polymorphisms with healthy individuals, the X/X genotype of rs7096206 was significantly higher in HIV-positive patients [21]. Sheng et al. found a higher prevalence of the heterozygous genotype with the B variant (rs1800450) in HIV-1-infected patients than in healthy controls, indicating that individuals with the B variant are more susceptible to HIV-1 infection [22].

However, such studies addressing host genetic susceptibility to HIV infection are limited. This study aimed to investigate the relationship of single nucleotide polymorphisms (SNPs) of the *TOLLIP* and *MBL2* genes with HIV infection in the Chinese Han population.

## Materials and methods

### Study population

This study was approved by the ethics committee of West China Hospital of Sichuan University [Approval No.: 932 (2019)]. The current research was done in accordance with the principles of the Declaration of Helsinki. The subjects understood the purpose and implementation plan of the study and signed informed consent forms. The patients’ legal representatives signed the consent for participants under 18 years.

The study consisted of 435 HIV seropositive patients (HSP) enrolled from outpatients attending the West China Hospital of Sichuan University and Neijiang Second People’s Hospital from January 2019 to December 2020. The inclusion criteria for case patients were as follows: 1) signed written consent; 2) ≥14 years old; and 3) the diagnosis of HIV infection based on laboratory tests. One thousand thirteen unmatched normal healthy controls with HIV seronegative (HSN) status were recruited for the present study from individuals attending the West China Hospital outpatient department for annual physical examination. All subjects were unrelated ethnic Han Chinese. The demographic characteristics of all subjects were obtained through a detailed questionnaire survey (Questionnaire Survey S1).

### Genotyping

Peripheral venous blood (4 ml) was collected in EDTA tubes (BD Vacutainers, Franklin Lakes, NJ, USA). DNA was extracted from whole blood using the AxyPrep DNA Blood kit (Axygen Scientific Inc., Union City, CA, USA) and then stored in a − 80 °C freezer for further analyses. Since our previous results indicate that the polymorphisms of the *TOLLIP* gene can affect the risk of pulmonary tuberculosis [15], this study also selected the three SNPs (rs5743899, rs3750920, and rs5743867) in the *TOLLIP* gene selected in previous studies. And two interesting SNPs (rs7096206 and rs1800450) of the *MBL2* gene related to the risk of HIV infection were selected [21, 22]. Finally, two SNPs in the *MBL2* gene (10q21.1) (rs7096206 in the promoter region and rs1800450 in the exon) and three SNPs in the *TOLLIP* gene (11p15.5) (rs5743899 in the intron, rs3750920 in the exon, and rs5743867 in the intron) were selected and analyzed. All selected SNPs were genotyped using a SNPscan Kit (Cat#: G0104, Genesky Biotechnologies Inc., Shanghai, China). The SNPscan assay technique is a rapid multiplex genetic screening system, and the basic principle of this technology is to recognize SNP alleles by using the high specificity of ligase binding reactions [23]. As a quality control measure, 5% of the duplicate samples were genotyped to check for concordance using the same process.

### Statistical analysis

Observed and expected genotype frequencies in the control group were compared by χ2 test to check deviation from Hardy–Weinberg Equilibrium. Odds ratios (ORs) and their 95% confidence intervals (CIs) were calculated to assess the risk conferred by a particular allele, genotype, and three genetic models (dominant, recessive, and additive models) using unconditional binary logistic regression, adjusted according to age and sex. *P*-values < 0.05 were considered to be significant. Statistical analysis was performed using SPSS software version 21 (SPSS, Chicago, IL, USA). The haplotype frequencies and linkage disequilibrium (LD) (using R^2^ as coefficients) between SNPs were calculated by using the SHEsis online software platform [24]. The multifactor dimensionality reduction (MDR) constructive induction algorithm was used to detect the gene-gene interaction. The MDR software and MDR permutation testing module are available from http://www.epistasis.org and http://sourceforge.net/projects/mdr/files/mdrpt. Power and sample size calculation software were used to calculate the power of our study [25].

## Results

### Patient characteristics and quality control results

In this case-control study, 435 HIV-infected patients and 1013 healthy controls were consecutively recruited. The demographic data and clinical characteristics of the study groups are summarized in Table 1. Significant differences were observed for age and sex between the case and control groups (*P* < 0.001 and P < 0.001, respectively).

**Table 1:** Characteristics of HIV infected patients and healthy controls

	Case group N(%)	Control group N(%)	P
Number	435	1013	
Mean Age	48.63 ± 15.159	41.24 ± 16.381	< 0.001
Gender			
Males	314 (72.18)	490 (48.37)	< 0.001
Females	121 (27.82)	523 (51.63)	

The success rate of genotyping ranged from 99.1–100%, and the accuracy of the 5% repetitive genotyping samples was 100%. In addition, there were no deviations from Hardy–Weinberg equilibrium in the control group (*P* > 0.05).

### Association between *TOLLIP* SNPs and HIV infection susceptibility

The allele and genotype frequencies among HIV-positive patients and healthy controls are shown in Table 2. The minor alleles of rs5743899 (C allele) and rs5743867 (G allele) in the *TOLLIP* gene showed a significantly increased risk of susceptibility to HIV infection (OR: 1.196, 95% CI: 1.026–1.393, *P* = 0.022; OR: 1.356, 95% CI: 1.175–1.564, *P* < 0.001, respectively). After adjusting for confounders, including age and sex, the rs5743899 C allele and rs5743867 G allele still showed a significantly increased risk of susceptibility to HIV infection (OR^a^: 1.231, 95% CI: 1.048–1.446, P^a^ = 0.011; OR^a^: 1.387, 95% CI: 1.191–1.615, P^a^ < 0.001, respectively). The rs5743899 CC genotype showed a significantly increased risk of susceptibility to HIV infection (OR^a^: 1.198, 95% CI: 1.008–1.424; P^a^ = 0.040) compared with genotype TT (Table 2). In the genetic model analysis, the SNP rs5743899 also showed a significantly increased risk of susceptibility to HIV infection in dominant and additive models (OR^a^: 1.317, 95% CI: 1.042–1.664; P^a^ = 0.021; OR^a^: 1.218, 95% CI: 1.034–1.435; P^a^ = 0.018, respectively) (Table 3). The rs5743867 GG genotype showed a significantly increased risk of susceptibility to HIV infection compared with the AA genotype (OR^a^: 1.222, 95% CI: 1.026–1.455; P^a^ = 0.012) (Table 2). As shown in Table 3, rs5743867 in the *TOLLIP* gene showed a significantly increased risk of susceptibility to HIV infection in all three genetic models (dominant, recessive, and additive models) (P^a^ = 0.01, P^a^ = 0.002 and P^a^ = 0.001, respectively). However, rs3750920 in the TOLLIP gene showed no significant association with HIV infection in any genetic model. No association was observed between the two selected SNPs in *MBL2* and susceptibility to HIV infection (Table 2 and Table 3).

**Table 2:** Frequency distribution of MBL2 and TOLLIP gene polymorphisms in HIV infected patients and healthy controls

Gene	SNPs	Allele/ Genotype	Case group N(%)	Control group N(%)	OR(95%CI)	P	OR^a^(95% CI)	P^a^
*MBL2 gene*	rs7096206(C > G)	Allele						
		C	714 (0.820)	1678 (0.831)	1	Reference	1	Reference
		G	156 (0.179)	340 (0.168)	0.998 (0.820–1.214)	0.983	1.050 (0.853–1.294)	0.644
		Genotype						
		CC	293 (0.673)	706 (0.699)	1	Reference	1	Reference
		GC	128 (0.294)	266 (0.263)	1.159 (0.902–1.490)	0.248	1.170 (0.899–1.521)	0.243
		GG	14 (0.032)	37 (0.036)	0.955 (0.697–1.308)	0.774	1.023 (0.734–1.426)	0.892
	rs1800450(C > T)	Allele						
		C	735 (0.846)	1667 (0.826)	1	Reference	1	Reference
		T	133 (0.153)	351 (0.173)	0.855 (0.699–1.045)	0.127	0.886 (0.716–1.096)	0.266
		Genotype						
		CC	312 (0.718)	689 (0.682)	1	Reference	1	Reference
		TC	111 (0.255)	289 (0.286)	0.848 (0.656–1.096)	0.208	0.860 (0.658–1.125)	0.271
		TT	11 (0.025)	31 (0.030)	0.885 (0.624–1.257)	0.495	0.901 (0.624–1.301)	0.578
*TOLLIP gene*	rs5743899(T > C)	Allele						
		T	504 (0.581)	1268 (0.628)	1	Reference	1	Reference
		C	362 (0.418)	750 (0.371)	1.196 (1.026–1.393)	0.022	1.231 (1.048–1.446)	0.011
		Genotype						
		TT	149 (0.344)	409 (0.405)	1	Reference	1	Reference
		CT	206 (0.475)	450 (0.445)	1.257 (0.979–1.613)	0.073	1.274 (0.980–1.655)	0.070
		CC	78 (0.180)	150 (0.148)	1.195 (1.012–1.410)	0.036	1.198 (1.008–1.424)	0.040
	rs3750920(C > T)	Allele						
		C	607 (0.700)	1391 (0.689)	1	Reference	1	Reference
		T	259 (0.299)	627 (0.310)	0.960 (0.816–1.129)	0.622	0.949 (0.799–1.126)	0.547
		Genotype						
		CC	207 (0.478)	486 (0.481)	1	Reference	1	Reference
		TC	193 (0.445)	419 (0.415)	1.081 (0.854–1.369)	0.515	0.999 (0.780–1.279)	0.994
		TT	33 (0.076)	104 (0.103)	0.863 (0.698–1.067)	0.174	0.861 (0.690–1.075)	0.186
	rs5743867(A > G)	Allele						
		A	503 (0.590)	1290 (0.639)	1	Reference	1	Reference
		G	349 (0.409)	728 (0.360)	1.356 (1.175–1.564)	< 0.001	1.387 (1.191–1.615)	< 0.001
		Genotype						
		AA	152 (0.356)	424 (0.420)	1	Reference	1	Reference
		GA	199 (0.467)	442 (0.438)	1.256 (0.978–1.612)	0.074	1.285 (0.0.989–1.669)	0.060
		GG	75 (0.176)	143 (0.141)	1.210 (1.023–1.430)	0.026	1.222 (1.026–1.455)	0.012

Abbreviation: *MBL2* mannose binding lectin 2; *TOLLIP* toll-interacting protein; *SNPs* single nucleotide polymorphisms; *OR* odds ratio; *CI* confidence interval; a, adjusted for age and gender

**Table 3:** Association between genetic models of gene polymorphisms and susceptibility to HIV infection

SNPs	Genetic model		OR(95%CI)	P	OR^a^(95% CI)	P^a^
rs7096206(C > G)	Dominant model	GG + GC vs. CC	1.068 (0.848–1.346)	0.576	1.108 (0.867–1.415)	0.414
	Recessive model	GG vs. CC + GC	0.729 (0.426–1.245)	0.247	0.846 (0.480–1.490)	0.562
	Additive model	2GG + GC vs.CC	1.036 (0.849–1.264)	0.729	1.078 (0.873–1.331)	0.485
rs1800450(C > T)	Dominant model	TT + TC vs. CC	0.840 (0.663–1.064)	0.148	0.867 (0.676–1.112)	0.262
	Recessive model	TT vs. CC + TC	0.826 (0.497–1.374)	0.462	0.897 (0.524–1.534)	0.691
	Additive model	2TT + TC vs. CC	0.857 (0.695–1.057)	0.149	0.882 (0.708–1.099)	0.262
rs5743899(T > C)	Dominant model	CC + CT vs. TT	1.273 (1.020–1.590)	0.033	1.317 (1.042–1.664)	0.021
	Recessive model	CC vs. TT + CT	1.213 (0.929–1.584)	0.157	1.263 (0.95–1.673)	0.104
	Additive model	2CC + CT vs. TT	1.200 (1.026–1.402)	0.022	1.218 (1.034–1.435)	0.018
rs3750920(C > T)	Dominant model	TT + TC vs. CC	1.019 (0.822–1.263)	0.862	0.971 (0.774–1.218)	0.799
	Recessive model	TT vs. CC + TC	0.802 (0.566–1.135)	0.213	0.856 (0.595–1.231)	0.402
	Additive model	2TT + TC vs. CC	0.953 (0.805–1.128)	0.574	0.932 (0.780–1.115)	0.442
rs5743867(A > G)	Dominant model	GG + GA vs. AA	1.424 (1.155–1.757)	0.001	1.469 (1.176–1.834)	0.001
	Recessive model	GG vs. AA+GA	1.461 (1.151–1.855)	0.002	1.500 (1.163–1.934)	0.002
	Additive model	2GG + GA vs. AA	1.282 (1.101–1.494)	0.001	1.305 (1.111–1.532)	0.001

Abbreviation: *SNPs* single nucleotide polymorphisms; *OR* odds ratio; *CI* confidence interval; *a* adjusted for age and gender

The R^2^ between rs5743899 and rs5743867 was 0.94 in these populations (Figure S1). To test whether the association of rs5743899 with HIV was dependent on rs5743867, the association was adjusted in the dominant model for rs5743867, in addition to age and sex. The results showed that rs5743899 was not independently associated with HIV infection. In other words, the association between rs5743899 and susceptibility to HIV infection may be due to its linkage disequilibrium (LD) and rs5743867.

### Haplotype analysis

Since no significant association with the *MBL2* gene was found, haplotype analyses were not performed using this gene.

Haplotype analyses were performed for the *TOLLIP* gene, and haplotypes with frequency < 0.03 were ignored. As shown in Table 4, three haplotypes, CCG, TTA, and TCA, were detected. We found that the CCG haplotype was significantly associated with an increased risk of HIV infection (OR^a^: 1.225, 95% CI: 1.039–1.444; P^a^ = 0.016). The other two haplotypes (TTA and TCA) showed no significant association.

**Table 4:** Haplotype analysis of TOLLIP gene SNPs in association with the risk of HIV infection

Haplotypes	Controls N(%)	HIV infected patients N(%)	OR^a^(95% CI)	P^a^
CCG	726 (35.9)	347 (40.8)	1.225 (1.039–1.444)	0.016
TTA	620 (30.7)	249 (29.2)	0.927 (0.777–1.025)	0.400
TCA	646 (32.0)	245 (28.8)	0.837 (0.703–0.997)	0.092

Abbreviation: *OR* odds ratio; *CI* confidence interval; *a* adjusted for age and gender

### Gene-gene interaction

MDR analysis was performed with all the tested SNPs to investigate potential genetic interactions associated with HIV infection. In this studied population, one SNP in the *MBL2* gene (rs1800450) and two SNPs in the *TOLLIP* gene (rs3750920 and rs5743867) formed the best interaction model with a testing balanced accuracy of 53.94% and cross-validation consistency of 6/10 (Table S1). However, none of the models were statistically significant using 1000-fold permutation testing (*P* > 0.05) (Table S1).

### Power analysis

We determined the sample size’s power for the five selected SNPs under the allelic model (Table S2). The results showed that our study has reasonable power (> 99%) to draw conclusions with OR of 2 or above.

## Discussion

Few data have evaluated the relationship between *TOLLIP* gene polymorphisms and susceptibility to HIV infection [7, 17]. Moreover, previous results were basic cell experiments and lacked extensive population-based sample genetic association studies. In the present study, two SNPs [rs5743899(T > C) and rs5743867(A > G)] in the *TOLLIP* gene were found to be significantly associated with HIV infection in the Chinese Han population. Further analysis showed that rs5743899 was not independently associated with HIV infection. We also observed an association of the *TOLLIP* haplotype with HIV infection. The CCG haplotype showed an increased risk for HIV infection in our study populations.

To our knowledge, this is the first report that provides evidence that *TOLLIP* gene polymorphisms are associated with susceptibility to HIV infection in Chinese Han populations. We found that two SNPs [rs5743899(T > C) and rs5743867(A > G)] in the *TOLLIP* gene were found to be significantly associated with HIV infection, while rs3750920 was not. Our results show that the rs5743899 and rs5743867 SNPs in the *TOLLIP* gene are in almost perfect LD in these populations. When adjusted in the dominant model for rs5743867, age and sex, we found that rs5743899 was not independently associated with HIV infection. That is, the association between rs5743899 and susceptibility to HIV infection may be due to its LD and rs5743867. A significant association with HIV infection was observed with the *TOLLIP* gene intron polymorphism rs5743867. A previous study found that the rs5743867 polymorphism is significantly associated with protection from sepsis in a Chinese Han population [14]. It has also been reported that the GG genotype of rs5743867 was associated with increased risks for pulmonary tuberculosis in the Chinese Han population [15]. The minor allele frequency of SNP rs5743867 was 0.409 in the control group in this study, which is similar to two previous studies conducted among the Chinese Han population; 0.39.5 in Song’s study [14], and 0.38 in Wu’s study [15]. Another SNP, rs3793964, in the *TOLLIP* gene was associated with an increased risk for leprosy and increased skin expression of *TOLLIP* and IL-1R antagonist [26]. Taken together, these studies demonstrated that polymorphisms in the *TOLLIP* gene were related to infectious diseases. It has been reported that rs5743867 in the *TOLLIP* gene is significantly associated with the levels of TNF-α and IL-6 [14], and rs5743899 is associated with increased IL-6 production [16]. This result indicated that the *TOLLIP* gene may affect mRNA expression. As an endogenous negative regulator of TLR signaling in the inflammatory response, *TOLLIP* can prevent cell signaling mediated by TLR2 and TLR4 by directly binding to TLRs or blocking IL-1 receptor-related receptor kinases [27]. Yang et al. proved that *TOLLIP* could inhibit HIV-LTR-driven gene expression by inhibiting the activation of NF-κB [17]. In addition, their results also indicate that *TOLLIP* plays a role in maintaining viral latency [17]. Li et al. found that *TOLLIP* can inhibit the activity of LTRs from multiple HIV-1 subtypes, and *TOLLIP* knockout in primary CD+ T cells can promote the activation of HIV from latent infection [7].

The MBL protein is encoded by the *MBL2* gene, which is a key molecule of the innate immune system and a Ca2 + −dependent C-type serum lectin mainly produced by the liver [28]. Early in vitro experiments showed that MBL could inhibit HIV infection by binding to HIV-1 gp120 glycoprotein [29]. MBL can also selectively bind to HIV-1-infected cells and inhibit viral infection of CD4+ T cell lines [30]. Variations in the *MBL2* gene encoding MBL may affect human susceptibility to HIV infection. Many genetic epidemiological studies have explored the relationship between *MBL2* gene mutation and HIV-1 infection, but the results are controversial and inconclusive [8, 9, 20–22, 31–33]. Tan et al. demonstrated a significant association between *MBL2* genotypes and MBL serum levels, suggesting an increased susceptibility to HIV-1 infection and disease progression with *MBL2* polymorphisms [32]. A statistical relationship was observed between *MBL2* gene polymorphisms and HIV-1 infection in the South Brazilian [21], Italian [20], and Chinese Han [22] populations. Boniotto et al. found that a 6 bp deletion at position − 328 was correlated with HIV-1 infection [20]. The frequency of the X/X genotype of rs7096206 was significantly higher in HIV-positive patients in South Brazil than in healthy individuals [21]. Another study found that the prevalence of the heterozygous genotype with the B variant (rs1800450) was higher in HIV-1-infected patients than in controls [22]. Other researchers did not find this association in Zambian [31], Chinese Han [8], Colombian [33], or white Spanish patients [10]. Consistent with Li et al. [8], our results in Chinese populations failed to find a significant association between *MBL2* gene polymorphisms and susceptibility to HIV infection, which indicated that gene polymorphisms in the *MBL2* gene might not be associated with HIV infection among this population. Considering the positive results in some other studies, there may be other variants not studied that may be associated with HIV infection. Further large-scale genome-wide association studies are needed.

Our research has some limitations. First, as a retrospective study, we lacked follow-up data, which may have limited our ability to analyze the association of *TOLLIP* gene SNPs with disease progression and outcome. Further prospective studies are needed. Second, this study included HIV uninfected healthy people as the control group, but we were unaware of their HIV exposure risk. As a result, we cannot analyze the genetic susceptibility of HIV infection in people who are also at risk of HIV exposure. Third, only two SNPs in *MBL2* were genotyped, and other potential functional variations may have been ignored. A large-scale genome-wide association study is needed in the future. Finally, the ethnic uniformity of this study was another limitation. This study only included the Chinese Han population. However, considering that there may be differences in SNP alleles and genotypes frequency of different ethnic groups, the conclusions of this data are only drawn to the Chinese Han population, and conclusions should be made cautiously when interpreting in other ethnic groups.

This study found that two SNPs (rs5743867 and rs5743899) in the *TOLLIP* gene associated with susceptibility to HIV infection were observed in the Chinese Han population, suggesting a potential role for the *TOLLIP* gene in susceptibility to HIV infection.

## Supplementary Information

**Additional file 1.**

**Additional file 2.**

## Data Availability

The datasets used and/or analyzed during the current study are available from the corresponding author on reasonable request.
