# Supplementary material for: Genetic association of TOLLIP gene polymorphisms and HIV infection: a case-control study
Source: BMC Infect Dis. 2021 Jun 21;21:590. doi: 10.1186/s12879-021-06303-4 (PMC8215734; doi:10.1186/s12879-021-06303-4)
Supplement: Supplementary file 1 — Additional file 1. [file 12879_2021_6303_MOESM1_ESM.doc]

Table S1 Multifactor dimensionality reduction analysis of SNPs and HIV susceptibility.

| Models | Testing balanced accuracy | Cross-validation consistency | P^a^ |
| --- | --- | --- | --- |
| rs1800450, rs3750920 | 0.5285 | 8/10 | 0.242 |
| rs1800450, rs3750920, rs5743867 | 0.5394 | 6/10 | 0.100 |

a P value for testing balanced accuracy using 1000-fold permutation test.

Table S2 Power of the study with different relative risks.

| Gene | SNP | Genetic model | MAF of control | Power % | | |
| --- | --- | --- | --- | --- | --- | --- |
|  |  |  |  | RR = 2 | RR = 3 | RR =4 |
| MBL2 | rs7096206(C>G) | Allele | 0.168 | 99.9 | 100 | 100 |
|  | rs1800450(C>T) | Allele | 0.173 | 99.9 | 100 | 100 |
| TOLLIP | rs5743899(T>C) | Allele | 0.371 | 100 | 100 | 100 |
|  | rs3750920(C>T) | Allele | 0.310 | 100 | 100 | 100 |
|  | rs5743867(A>G) | Allele | 0.360 | 100 | 100 | 100 |

Abbreviation: SNP, single nucleotide polymorphism; MAF, minor allele frequency; RR, relative risks.

Figure S1 Pair-wise LD between SNPs in *TOLLIP* gene was evaluated by R^2^ statistics.

Questionnaire Survey S1: Questionnaire Survey

**Questionnaire Survey**

|  |  | **General Information** | | | | | | | | |
| --- | --- | --- | --- | --- | --- | --- | --- | --- | --- | --- |
| Name | | |  | | | | Registration number | |  | |
| Sex | | |  | | | | ID number | |  | |
| Race | | |  | | | | Contact Number | |  | |
| Education degree | | |  | | | | Marital status | |  | |
| Occupation | | |  | | | | Affiliation | |  | |
| Present address | | |  | | | | | | | |
|  |  | **Patient management** | | | | | | | | |
| Patient classification | | | | | 1 New patient 2 Return 3 immigration 4 Other: | | | | | |
|  |  | **Epidemiological data** | | | | | | | | |
| HIV exposure | | | 1 Blood transmission 2 Heterosexual contact 3 Same-sex sexual contact 4 Mother-to-child transmission | | | | | | | |
|  |  | **Symptom** | | | | | | | | |
| Fever | | | 1No 2 > 2 weeks 3 < 2weeks Maximum body temperature ( ℃) | | | | | | | |
| Pharyngalgia, weakness | | | 1Yes  2 No | Nausea, vomiting, diarrhea | | 1Yes  2 No | | Persistent generalized lymphadenopathy | | 1Yes  2 No |
| Description of other symptoms | | |  | | | | | | | |
|  |  | **Past illness** | | | | | | | | |
| Diabetes | | | 1No 2Yes Types of diabetes: type 1 type 2 special type: | | | | | | | |
| Liver disease | | | 1No 2Yes （ description: ） | | | | | | | |
| History of kidney disease | | | 1No 2Yes （ description: ） | | | | | | | |
| Chronic bronchitis | | | 1No 2Yes （ description: ） | | | | | | | |
| Other medical history | | |  | | | | | | | |
