# Supplementary figures and images for: Genetic association of TOLLIP gene polymorphisms and HIV infection: a case-control study
Source: BMC Infect Dis. 2021 Jun 21;21:590. doi: 10.1186/s12879-021-06303-4 (PMC8215734; doi:10.1186/s12879-021-06303-4)

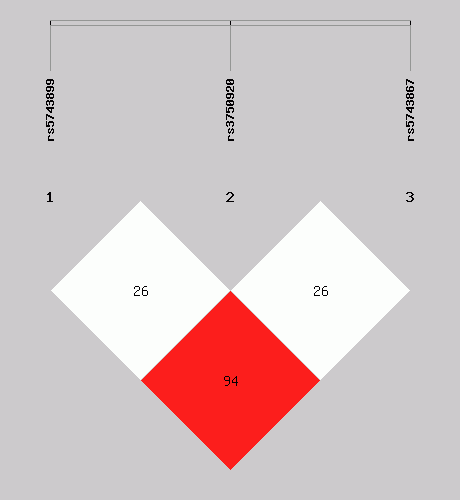

Supplement: Supplementary file 2 — Additional file 2. [file 12879_2021_6303_MOESM2_ESM.tif]
